# Endoscopic and Pathologic Resolution of Chronic Nonsteroidal Anti-Inflammatory Drug-Induced Diaphragm-Like Colonic Strictures and Ulceration

**DOI:** 10.1155/2018/7824081

**Published:** 2018-05-31

**Authors:** Behdod Poushanchi, Hiren Vallabh, Justin Kupec

**Affiliations:** ^1^Department of Medicine, West Virginia University, Morgantown, WV, USA; ^2^Section of Digestive Diseases, West Virginia University, Morgantown, WV, USA

## Abstract

The chronic use of nonsteroidal anti-inflammatory drugs (NSAIDs) has steadily increased and, as a result, adverse effects have become more common. Isolated case reports have documented diaphragm-like colonic strictures and ulceration as the result of NSAID use. We report a unique case of this rare side effect with documented endoscopic and histologic healing of multiple proximal diaphragm-like colonic strictures and ulceration months after simple discontinuation of NSAID therapy.

## 1. Introduction

In the United States, the chronic use of nonsteroidal anti-inflammatory drugs (NSAIDs) has surfaced as a growing problem. Per a 2010 report, 43 million adults (19.0%) took aspirin at least three times per week for more than 3 months (i.e., regular users), and more than 29 million adults (12.1%) were regular users of NSAIDs, an overall increase of 57% and 41%, respectively, from 2005 [1]. Long-term use of this pharmacotherapy is not recommended, and, in the aging adult, adverse effects have become critical [2]. Peptic ulcer disease is a well-known complication of this disease, and this may result in more than 100,000 hospital admissions and more than 7,000 deaths [3]. Less is known about NSAID-induced colopathy, with a range of case reports detailing a spectrum from acute inflammation and ulceration to chronic strictures and fibrosis [4].

## 2. Case Report

**Figure 1:**
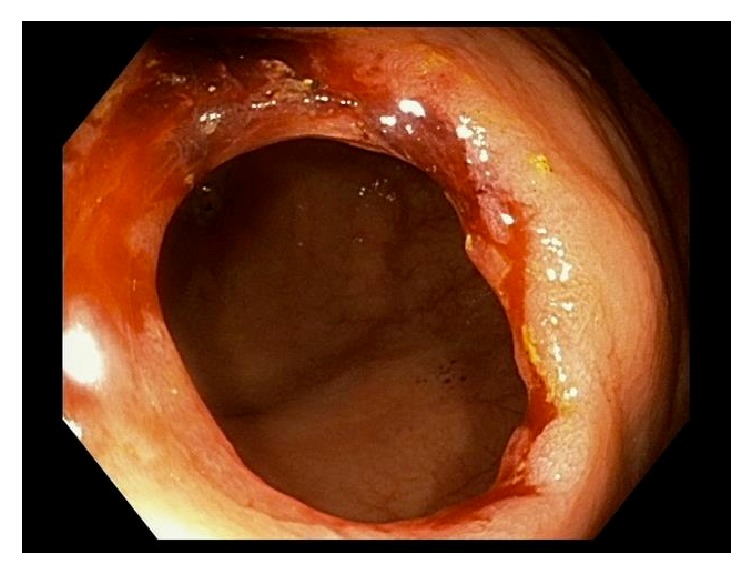
Endoscopic view of the ascending colon demonstrating a diaphragm-like stricture with scarring and ulceration.

**Figure 2:**
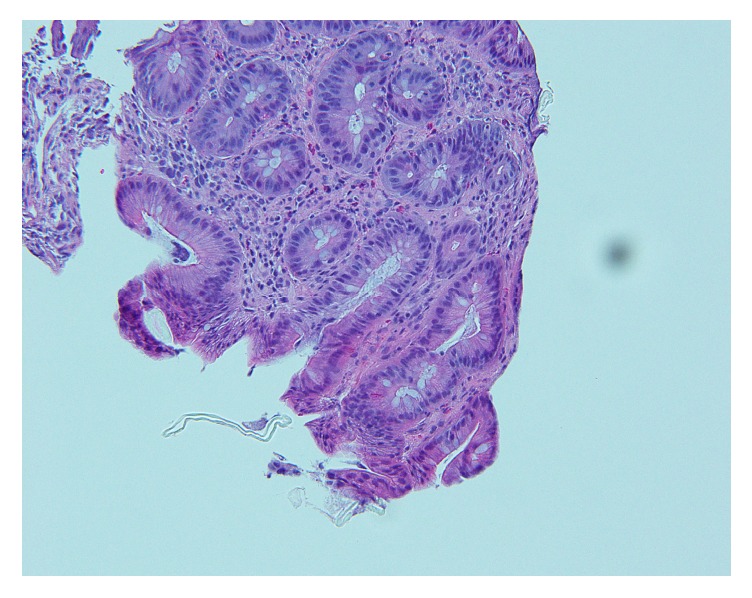
Ascending mucosal biopsy demonstrating ulceration and active inflammation.

**Figure 3:**
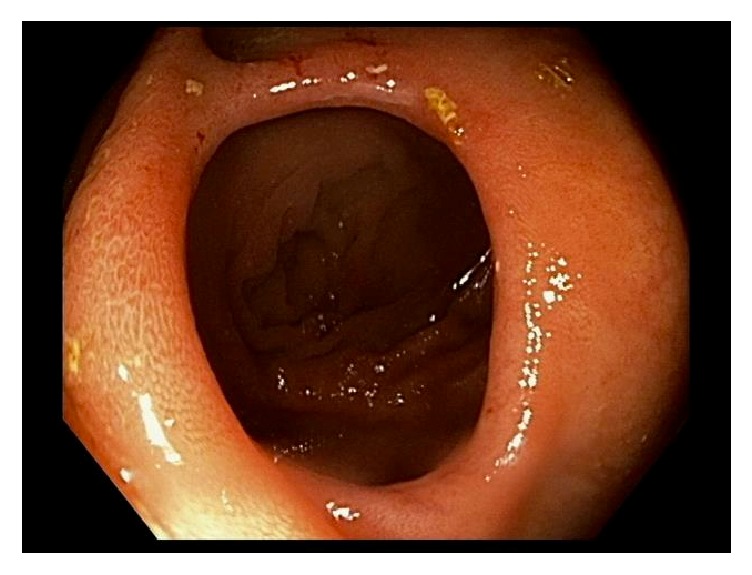
Endoscopic view of the ascending colon with resolution of diaphragm-like stricture, scarring, and ulceration.

**Figure 4:**
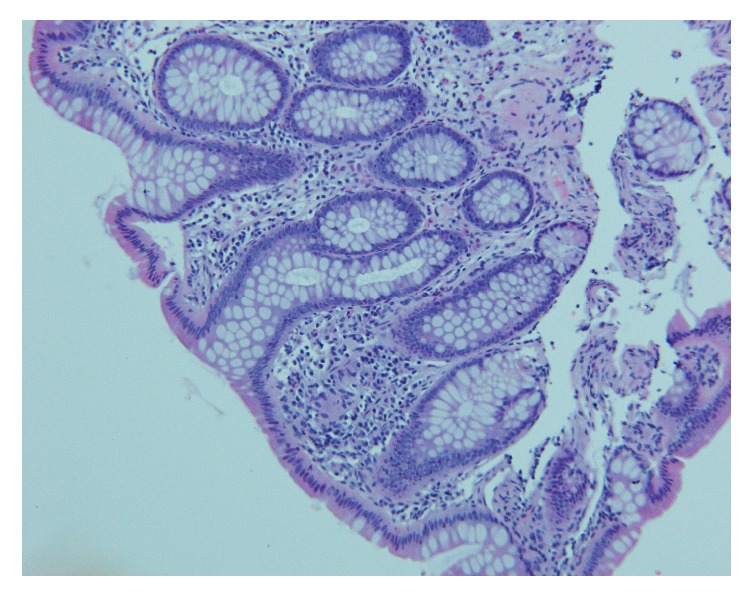
Ascending mucosal biopsy demonstrating resolution of ulceration and inflammation.

A 66-year-old woman presented for an outpatient colonoscopy for evaluation of six months of iron deficiency anemia, diarrhea, and rectal bleeding. Her history was significant for gastroesophageal reflux disease and chronic low back pain, on twice-daily naproxen. Serologic studies were notable for hemoglobin of 7.4 g/dL and a mean corpuscular volume of 70.6 fL. Colonoscopy demonstrated four diaphragm-like strictures, with scarring and ulceration, interspaced between normal mucosa in the cecum and ascending colon (Figure 1). Biopsies obtained from the cecal and ascending strictures revealed ulceration, acute inflammation, and reactive changes (Figure 2). The patient was instructed to discontinue naproxen and avoid all other NSAIDs. Patient was subsequently scheduled for a repeat colonoscopy in three months to monitor for resolution. Subsequent colonoscopy revealed both endoscopic (Figure 3) and pathologic resolution (Figure 4) of the diaphragm-like proximal colonic strictures.

## 3. Discussion

Adverse effects in patients that chronically utilize NSAIDs are well established. This includes gastric and small bowel inflammation, ulceration, and strictures which can be complicated by pain, bleeding, or perforation. Colopathy related to NSAIDs can include nonspecific colitis, colonic ulceration, relapse of inflammatory bowel disease, complications of diverticular diseases including hemorrhage, fistula formation, and perforation, and, as in our case, diaphragm-like colonic strictures and ulceration [5]. There are only isolated case reports supporting the relationship between NSAIDs and diaphragm-like colonic strictures and ulceration. The first case of colonic strictures with ulceration was reported in 1989 by Sheers and Williams [6]. Most diaphragm-like colonic strictures reported in the literature are limited to the right colon (79% of cases), per review by Penner et al [7].

Prior to appearance of symptoms related to adverse events related to NSAIDs, the span of exposure of NSAIDs has ranged from 2 days to 12 years, with median of 3 months [5]. Classic symptoms, which are often nonspecific, can include abdominal discomfort, diarrhea, gastrointestinal bleeding, or weight loss [5]. In our patient, retrospective questioning revealed five-year duration of naproxen use prior to developing symptoms.

While symptoms and adverse events remain well documented, the pathophysiology remains speculative at this time. A possible theory related to NSAID-induced colopathy starts with topical biochemical damaging action, an increase in mucosal permeability [8]. An interesting exception to conventional NSAIDs and increased intestinal permeability are aspirin and nabumetone. The theory regarding these pharmacotherapeutic agents may relate to their site of absorption and lack of excretion in the bile, both of which lead to decreased exposure of the drug to the mucosa [9]. After increased permeability and movement into the cells, there is uncoupling of oxidative phosphorylation, efflux of ions, and resultant oxygen radical damage [10]. Additionally, with the increased permeability, cells are exposed to proteolytic enzymes and bacterial invasion [5]. Neutrophil chemotaxis can lead to further damage and eventual ulcer and fibrotic stricture formation.

The obvious first step in patients with NSAIDs-related colopathy appears to be discontinuation of the offending agent. The review by Penner et al. showed nine patients with NSAIDs-related colonic strictures who were managed conservatively by discontinuing the NSAIDs, of which one patient had no follow-up, three became asymptomatic, and four had endoscopic or radiological follow-up that showed no change to the lesions after two weeks, five weeks, 18 months, and two years, respectively [7]. To the best of our knowledge, our case report presents a unique perspective in management of NSAIDs-related diaphragm-like strictures and ulceration, in which simple discontinuation of the offending agent revealed both clinical improvement and endoscopic and histologic healing.

If conservative management fails with simple discontinuation of the NSAIDs, a spectrum of therapies, from prednisone to endoscopic management with balloon dilatation to segmental colectomy, has been proposed for management of diaphragm-like colonic strictures [5, 11].

This case report does not prove a cause-and-effect relationship and colonic healing may have been coincidence. That being said, we propose that physicians strongly consider conservative management with pharmacotherapy cessation throughout the clinical decision-making process. The most effective management may be the simplest. As exhibited by our case, early cessation of the offending agent, in this case naproxen, revealed both endoscopic and histologic resolution of the multiple proximal diaphragm-like colonic strictures and ulcerations. This strategy may be overlooked in an era of developing endoscopic intervention, but it invokes the thought that, sometimes, less is more.
